# Clinical trainee performance on task‐based AR/VR‐guided surgical simulation is correlated with their 3D image spatial reasoning scores

**DOI:** 10.1049/htl2.12066

**Published:** 2024-01-08

**Authors:** Roy Eagleson, Denis Kikinov, Liam Bilbie, Sandrine de Ribaupierre

**Affiliations:** ^1^ Artificial Intelligence and Software Engineering The University of Western Ontario London Canada; ^2^ Clinical Neurological Sciences London Health Sciences Centre Ontario Canada

**Keywords:** augmented reality, computer based training, graphical user interfaces, interactive devices, man‐machine systems, surgery, user interfaces, virtual reality, visual perception

## Abstract

This paper describes a methodology for the assessment of training simulator‐based computer‐assisted intervention skills on an AR/VR‐guided procedure making use of CT axial slice views for a neurosurgical procedure: external ventricular drain (EVD) placement. The task requires that trainees scroll through a stack of axial slices and form a mental representation of the anatomical structures in order to subsequently target the ventricles to insert an EVD. The process of observing the 2D CT image slices in order to build a mental representation of the 3D anatomical structures is the skill being taught, along with the cognitive control of the subsequent targeting, by planned motor actions, of the EVD tip to the ventricular system to drain cerebrospinal fluid (CSF). Convergence is established towards the validity of this assessment methodology by examining two objective measures of spatial reasoning, along with one subjective expert ranking methodology, and comparing these to AR/VR guidance. These measures have two components: the speed and accuracy of the targeting, which are used to derive the performance metric. Results of these correlations are presented for a population of PGY1 residents attending the Canadian Neurosurgical “Rookie Bootcamp” in 2019.

## Graphical abstract

This paper describes a methodology for the assessment of training simulator‐based computer‐assisted intervention skills on an AR/VR‐guided neurosurgical procedure. Convergence is established towards the validity of the assessment methodology by examining two objective measures of spatial reasoning, along with one subjective expert ranking methodology, and comparing these to AR/VR guidance.

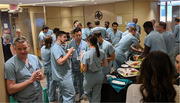

## 1 INTRODUCTION

**FIGURE 1:**
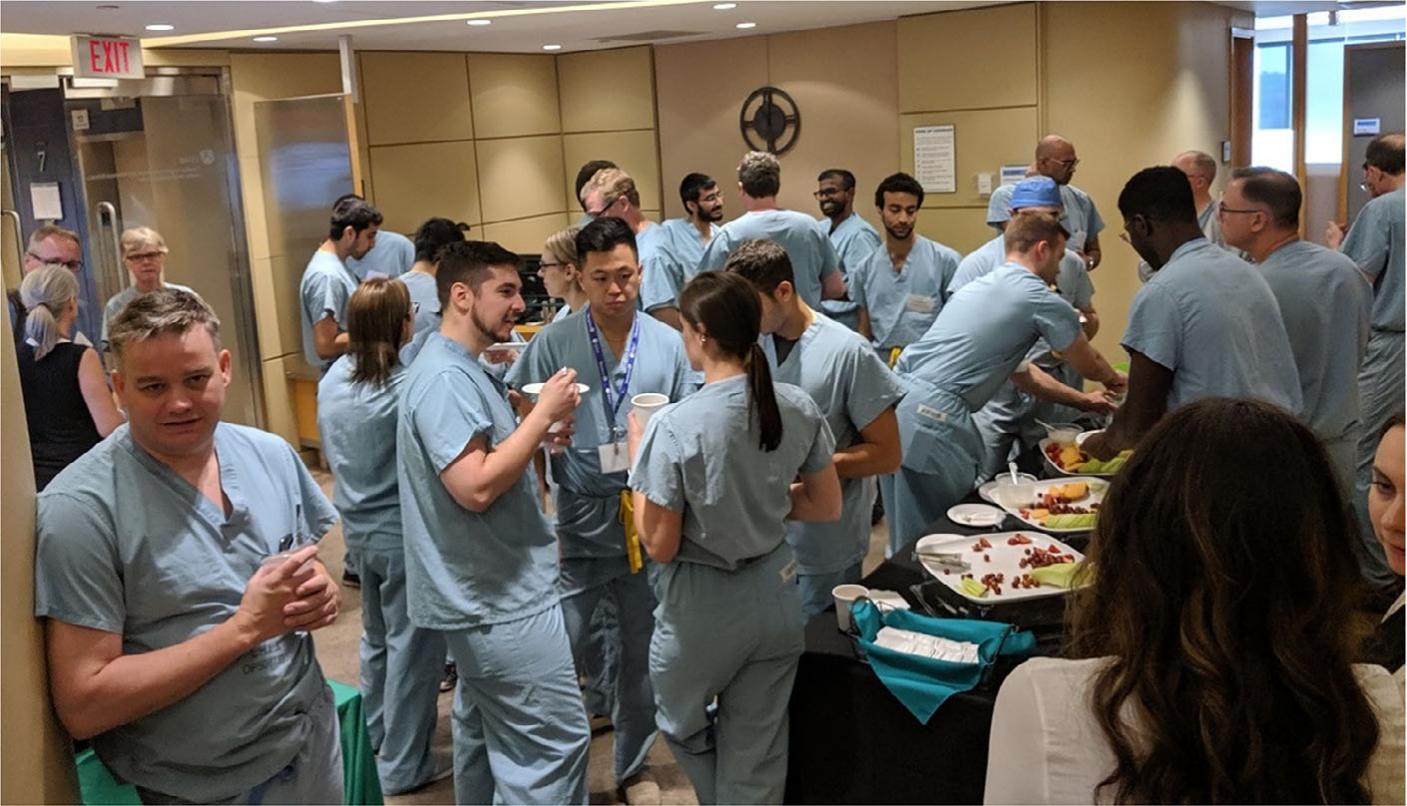
25 PGY1 residents attended the Canadian Neurosurgical ‘Rookie Bootcamp’ in 2019, hosted at the Canadian Surgical Technologies and Advanced Robotics (CSTAR) Centre.

At one level, this paper describes experiences and lessons learned while conducting a neurosurgical training ‘bootcamp’ (see Figure 1) at canadian surgical technologies and advanced robotics (CSTAR), in Canada in 2019. This paper also includes a description of the overall course objectives and descriptions of the kinds of training techniques used at each of the AR/VR simulator‐based [1, 2] training stations that were scheduled for the 53 trainees. This paper also describes the kinds of simulations, with particular emphasis on the methodologies used for gathering data, the systematic evaluative procedures in place at each station, and the results analysed from these data; gathered from 53 PGY1 resident trainees (Figure 1). Moreover, at another more philosophical level (more interesting for the AECAI audience) this paper carries a narrative and sets up an initial salvo that opens opportunities for discussion at the workshop about what should be methodologies for evaluating the effectiveness of simulation‐based training involving augmented environments for computer‐assisted interventions, and especially for neurosurgical procedures [3].

### 1.1 Canadian neurosurgery PGY1 bootcamp training

The Canadian Neurosurgery Society has sponsored, in collaboration with the Neurosurgery Specialty Committee of the Royal College of Physicians and Surgeons, since 2013 [4, 5] an intensive training camp for first‐year post‐graduate residents (PGY1). The ratio of instructors to trainees is approximately 1:2, and the trainees can expect to experience a full gamut of training experiences ranging from didactic to pragmatic, from lectures to hands‐on low‐fidelity box simulators.

This paper considers, a subset of these training exercises—ones which involve AR/VR‐based simulator training scenarios. In particular, these will involve extra‐ventricular drain (EVD) placement or alternately, endoscopic third ventriculostomy (ETV). Both of these procedures have a main surgical phase that involves the targeting of brain ventricles.

### 1.2 The red herring: Training simulator realism and face validity

**FIGURE 2:**
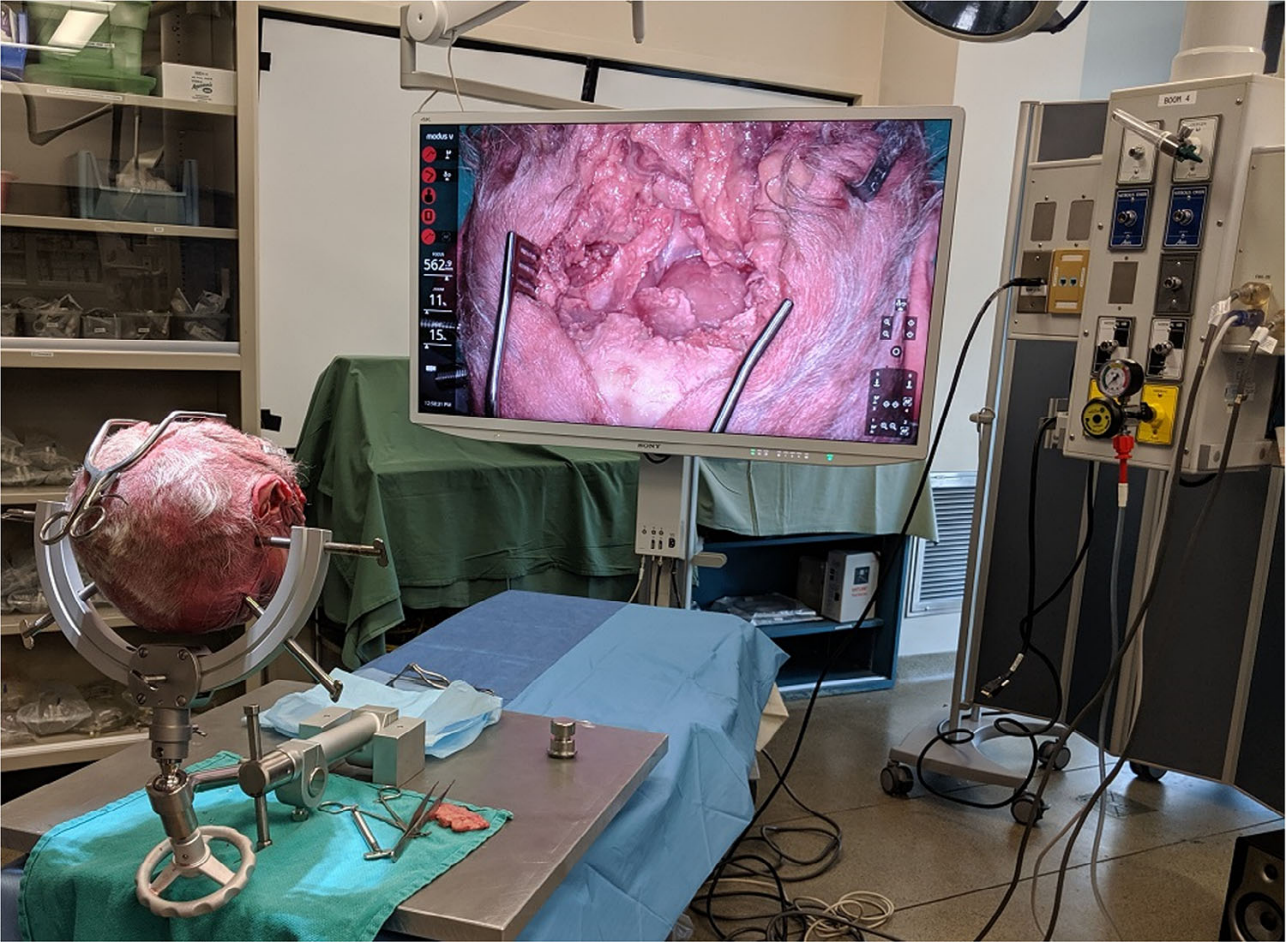
Surgical simulators can have varied ‘realism,’ to be sure. This one, in particular, is extremely realistic but has no intrinsic curriculum; a grim demonstration the ‘realism’ is not the key issue for ‘face validity.’ Most simulators have a programmed curriculum workflow, and so in fact, it is the validity of the simulator's assessment *metrics* that can be studied.

When considering the evaluation of a surgical simulator, one often considers the amount of ‘realism’ that can be designed for the simulation. This paper will argue that this is a red herring, and in fact, it is generally confused with ‘face validity.’ [6] For example, if you present a realistic wound to a trainee without a task and therefore without a test of performance (as if you were left in the room pictured in Figure 2), then its “face validity” is zero. Face validity means: ‘on the face of it,’ (i.e., *prima facia*): would an expert consider the testing evaluation to be a reasonable one? Similarly, consider an AR/VR‐based simulator that displays realistic views of a simulated neurosurgical case. Without a test, ‘realism’ has nothing to do with the ‘face validity’ of that simulator. [7] Accordingly, with a ‘fully realistic’ VR environment, a trainee might just as well be facing a cadaver head without any instructions, if they have no curriculum. To be sure, ‘face validity’ estimates *must* be estimated by domain experts based on an assessment of the evaluation *‘metrics’* for the explicit tasks that have been chosen from the curriculum (not from naive estimates of the ‘realism’ of a simulator). [8] To make the point sharper: Do the measures of performance on the simulator seem to an expert to be *reasonable* measures of their performance as related to the real task? *That* is ‘face validity.’

### 1.3 The real problem: How can we move towards integrating the surgical ‘process model’ with the ‘evaluation methodology?’

**FIGURE 3:**
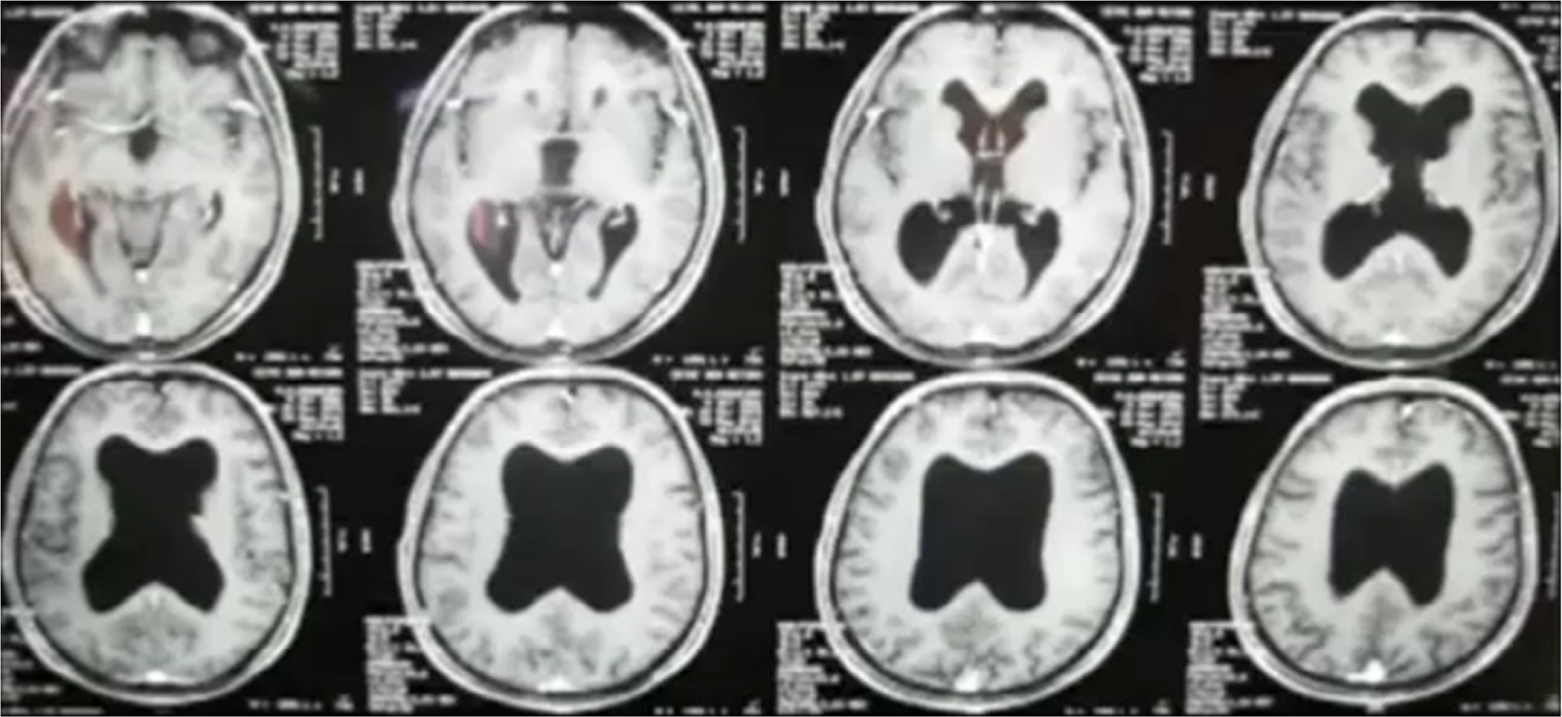
The skilled task being trained is to view a set of CT slices, and then, on the basis of reasoning about those views, to plan a point of entry on the skull and then introduce the EVD into the ventricular system.

**FIGURE 4:**
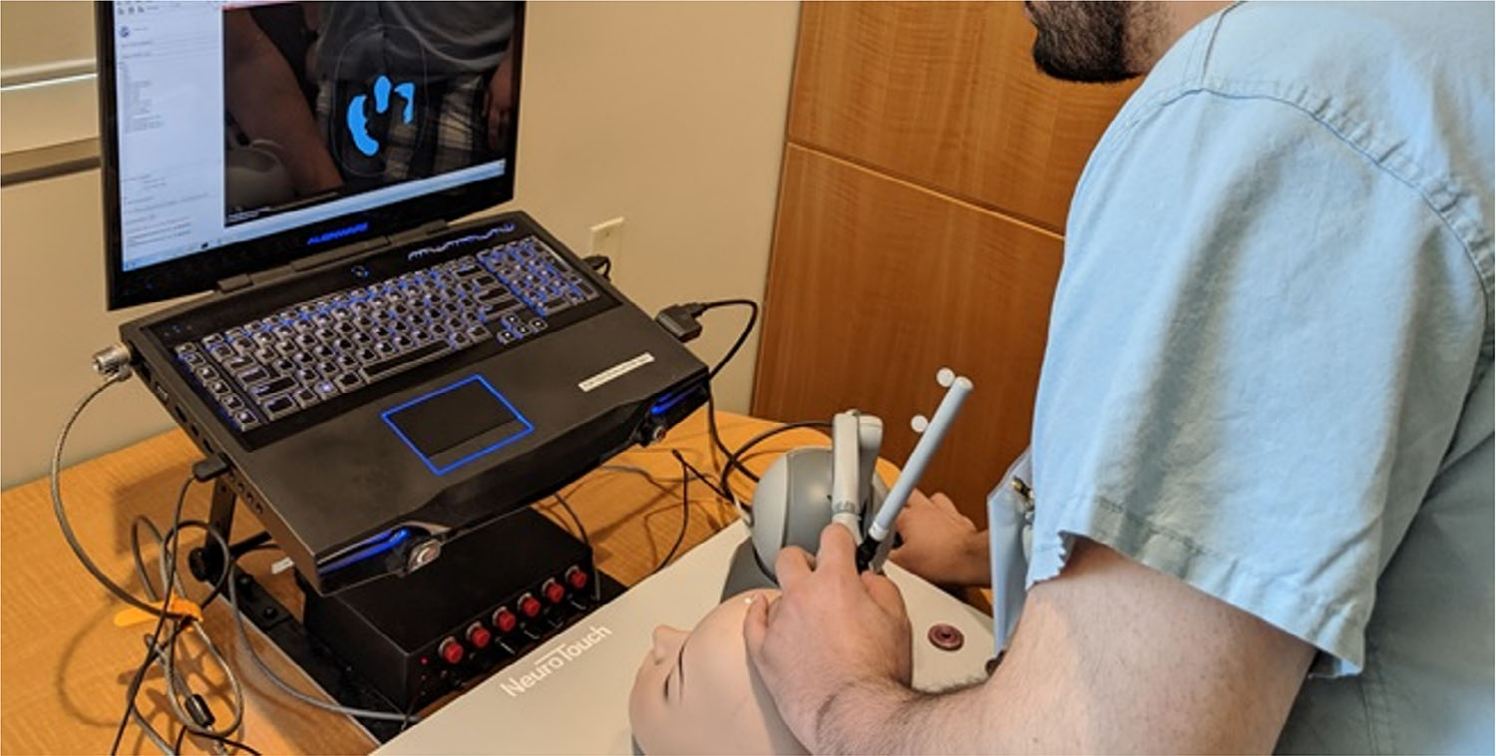
Once the trainee has reasoned about the point of entry and the location of the target, they must move the surgical tool through their estimated 3D entry position along an estimated 3D orientation and depth.

We will be examining a set of procedures that involve targeting neuroanatomical structures within the brain. The trainees will be performing this after viewing the patient's preoperative scans (Figure 3) using mixed reality for surgical guidance [9]. After reviewing them, they must form a mental representation of the target within the patient. The trainees will be required to learn how to transform that 2D information into 3D in order to visualize a location for entry (on the surface of the skull), along with a 3D direction vector and a depth of entry into the brain (with the experimental apparatus shown in Figure 4).

This central phase of the surgical procedure is quite well‐posed, and so measures of the 3D position, the 3D orientation, and the depth of entry are all well‐specified quantities that can be gathered on time‐stamped log file entries. These log files can be examined line‐by‐line in order to form assessments that facilitate inter‐ and intra‐trainee evaluations. Individuals can be compared across their training group and can be compared to data gathered from expert neurosurgeons.

## 2 ESTIMATES OF PERFORMANCE IN LOCALIZATION TASKS

In this section, we will expose the numerical relationship between the rankings assigned by clinical experts (neurosurgery consultants) who are training PGY1 residents and the mathematically well‐posed distance metric. A consultant neurosurgeon was asked to rank the ventricle targeting on a scale of 1 to 6. In the same spirit as the mathematical distance metric derived purely geometrically, a subjective ranking of targeting performance was assigned to each targeting trial by a consulting neurosurgeon.

The subjective scores were assigned such that, ‘if the targeting was placed well within the ventricle, with a good approach angle,’ then the score given was 1. If the targeting was in the ventricle but at a poor angle or inside but close to the ventricle wall, then the score was 2. If the targeting was slightly outside the ventricle, then the score was 3. If the targeting was outside the ventricle but with a poor approach angle, the score was 4. And finally, if the targeting was a wide miss, the score was 5 or 6, depending on whether the approach angle was good or not, according to expert subjective scoring.

**FIGURE 5:**
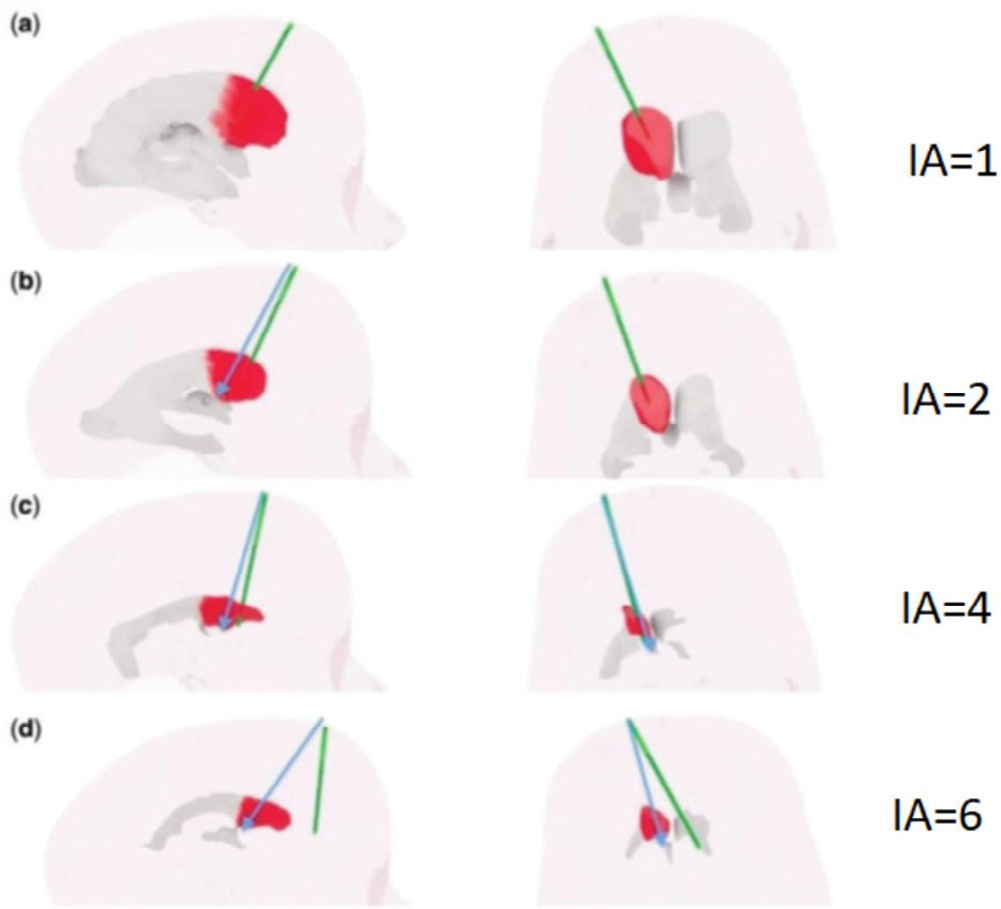
After each trial, for each participant, the targeting of the randomly presented ventricular system is displayed as a green line as feedback to the participant (with a blue line showing an ideal trajectory). A screenshot of each trial is saved, and it is subsequently ranked by an expert neurosurgeon. Sample rankings of the index of accuracy (IA) assigned by the neurosurgical expert are shown.

Just as reading a length measure from a ruler can be argued as being either ‘subjective’ or “objective,” depending on how much trust is placed on the observer, then so can an expert's ranking be considered objective, so long as there can be a principled functional relationship from their scores to a metric function. A set of sample expert‐assigned subjective scores [10] is illustrated in Figure 5, and full results and analysis are in the Results section.

## 3 RELATIONS BETWEEN OBJECTIVE METRICS AND EXPERT RANKINGS

Purely geometrical analysis can lead to the implementation of purely objective metrics. However, when considering patient‐specific anatomical structures, objective metrics can be infeasible to formulate or compute. Fortunately, subjective metrics, which can be assigned by experts, can be articulated in a way that maintains consistency with the well‐formed geometrical objective metrics. This consideration will then lead us to a broader and more over‐arching discussion about the parallels between objective metrics and subjective scores as we consider the unavoidable trade‐off between ‘internal validity versus external validity.’’

### 3.1 Construction of a purely geometrical metric

**FIGURE 6:**
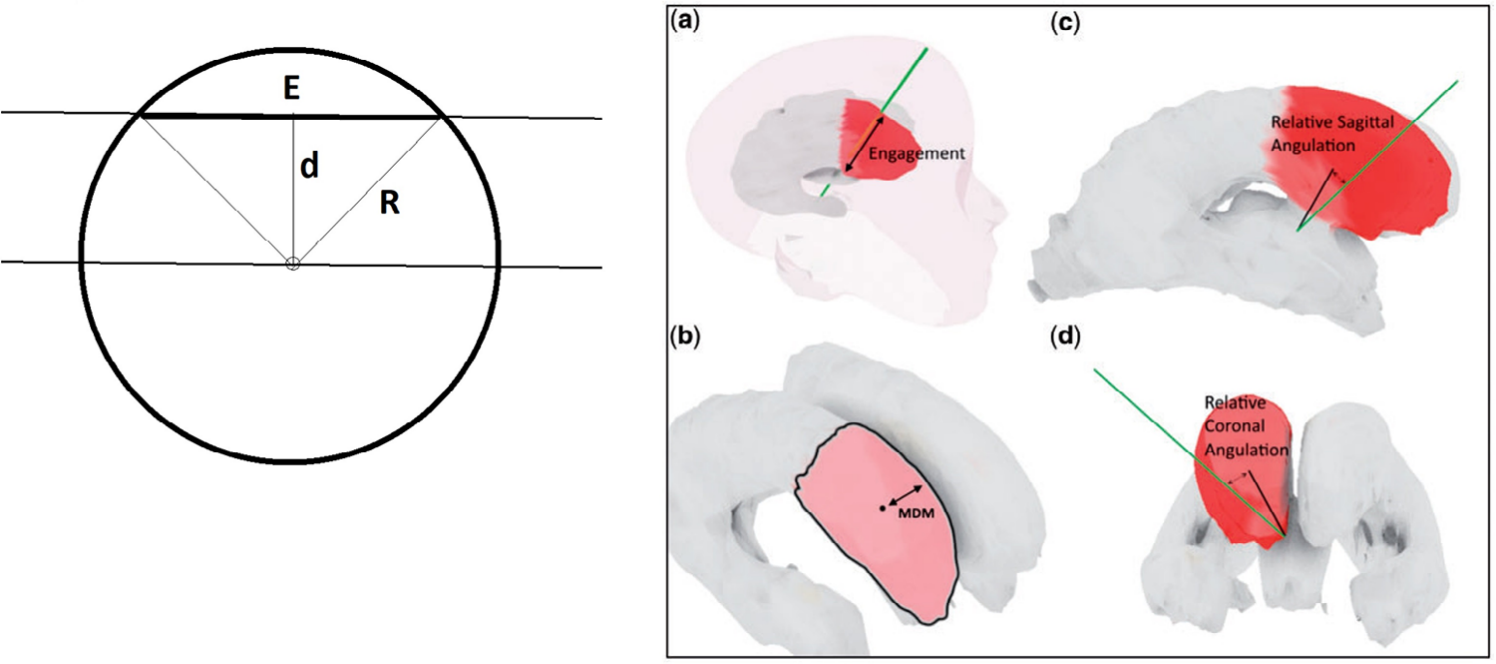
‘Engagement’ is a natural objective measure of the success of targeting a ventricle, but in addition, we can show the relationship between ‘engagement’ and a distance metric, which is then in a form to be used as an error measure.

Consider Figure 6, which shows a number of measures proposed from the literature that can be used to estimate the accuracy of ventricle targeting: (a) ‘Engagement,’ (b) MDM (distance between point and closest ventricle wall), (c) angulation in the sagittal plane and (d) angulation in the coronal plane. We take a moment here to justify why ‘engagement’’ may be a very appropriate measure for EVD placement performance and relate it to the classical Euclidean distance metric.

In principle, we would like to construct a metric which is such that if the surgeon places the catheter anywhere inside the ventricle, the task has been performed with ‘utility’ (i.e., in a binary go/no‐go sense)—yet questions can still arise about a quantitative score to be attributed to the procedure. Consider the case where the ventricle has been missed: heuristically, being far away from the ventricle should be associated with an error measure, and so the Euclidean distance metric (distance from the centre of the ventricle) is an appropriate measure. However, inside the ventricle, the metric should be ‘close to zero’ anywhere in the centre of the ventricle, but smoothly increasing as approaching the ventricle wall. We will now extend this heuristic to mathematical formalism as follows: Consider ‘E’ to be the engagement, and so if the target volume was a sphere of radius R, we could use E to form a very well‐formed distance metric. Through simple geometry, since in the left diagram in Figure 6, $R^{2}={\left(E/2\right)}^{2}+d^{2}$, we have the following expression: $D=R-\sqrt{R^{2}-d^{2}}$.

**FIGURE 7:**
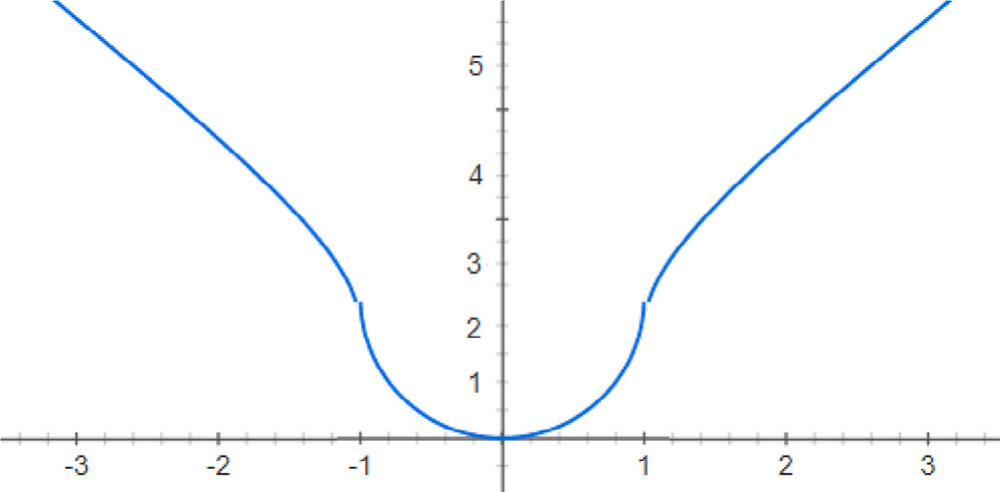
Functional relationship between normalized objective distance metric D (domain) and expert ranking “index of accuracy” (range), illustrating the central Lorentz transformation within the sphere of radius = 1.

This distance metric has very interesting and satisfying properties. By observation, when the trajectory is through the centre, giving maximal ‘engagement,’ then D = 0 (perfect accuracy). As d increases by a small amount, D increases by a much smaller amount, corresponding to the interpretation, “if the user has targeted in the vicinity of the central part of the volume, then the distance D is very close to zero.” As d is increased more and more, towards *R*, then the distance metric *D* begins to increase rapidly towards *R* (in fact, 1−*D*/*R* is similar to the Lorentz transformation: $\sqrt{1-d^{2}/R^{2}}$), shown in Figure 7.

When d = R, then D = R, but then subsequently, as the targeting ‘just misses’ the ventricle but is just outside the ventricle wall, then the distance metric D switches to an imaginary number, which serves as a ‘flag’ to indicate that the targeting is outside the ventricle, at a distance greater than R from the centre. Mathematically, just outside the ventricle wall, D= iR. Then, as we consider what happens as d increases to a ‘wide miss’ of the ventricular system, so that d is much larger than R, the distance metric D approaches that value as an imaginary number: D⟶id. The absolute value of D increases monotonically (which is a necessary property of a distance metric). To summarize, D is close to zero anywhere in the vicinity of the central region of the volume, D is most sensitive to changes of position at the edge of the volume, whereupon D = R when d = R, and then, D is approximately d, far away from the volume, and in all of these cases, D increases monotonically as the classical Euclidean distance. Accordingly, one can see that this is an objective and well‐posed distance error metric.

## 4 EVALUATION OF BASELINE SPATIAL REASONING SKILLS OF PARTICIPANTS

Our over‐arching commentary in this paper is intended to prompt discussion about whether the evaluation of the performance of a participant on a simulator should follow the model of expert rankings and subjective assessments—or whether it should be modeled more on the paradigms of experimental psychology and cognitive science. In the sequel, we will argue that “construct validity” can only be attained asymptotically after effortful iterative convergence between both approaches. To begin, we examine a classical methodology for establishing a baseline of user performance for spatial reasoning.

### 4.1 3D spatial reasoning from clinical 2D images

It is not controversial to state that the task of observing 2D slices of neuroanatomical structures and developing a 3D internal representation of those structures forms the foundation for the planning phase of this procedure. The internal representation allows the surgeon to form a plan for burr hole placement, and to move in a way that allows them to introduce the EVD from that location on the skull, at an angle that will allow the tool to intersect the ventricular system.

What is controversial, however, is the nature of this skill: from an information‐processing perspective, how are the input images being transformed into a motor plan and an action? It is this skill that is the construct we are trying to measure. Accordingly, this consideration leads to the question: Is this form of spatial reasoning a bottom‐up process? (learnable by trial and error, analogous to some deep‐learning prescription) or is it a top‐down cognitive process that can be learned through instruction and debriefing? To address this question, we make use of our evaluation methodology to explore the nature of spatial reasoning, within the context of what some would call “mental rotation.”

### 4.2 Objective methodology for evaluating 3D spatial reasoning from 2D images

We have implemented a computer‐based test that makes use of the same set of stimuli introduced by Roger Shepard [11] and Jacqueline Metzler in 1971. For each trial, participants view a pair of randomly‐selected images, which are 2D presentation of 3D objects, which are simple block shapes. The pairs are systematically randomized so that half of the time the items are “Same,” and the other half of the trials are “Different.” The participants press a key ‘s’ or ‘d,’ and the response is recorded in addition to the response time.

### 4.3 Results and analysis

**FIGURE 8:**
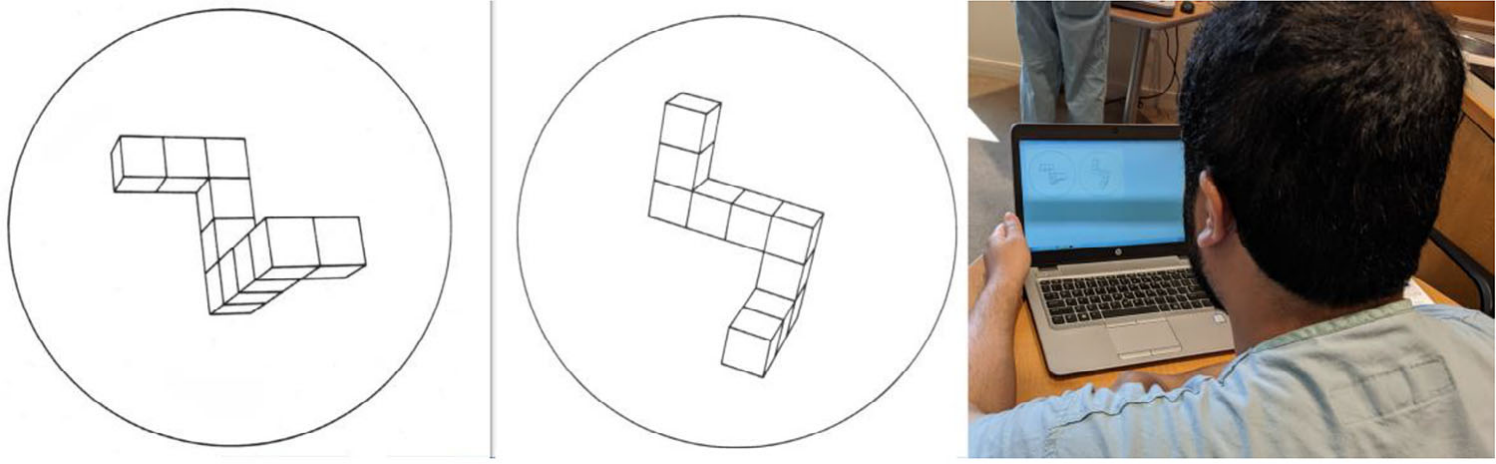
After each session on the EVD simulator, participants were tested using the stimuli from Shepard and Metzler's classical task.

**FIGURE 9:**
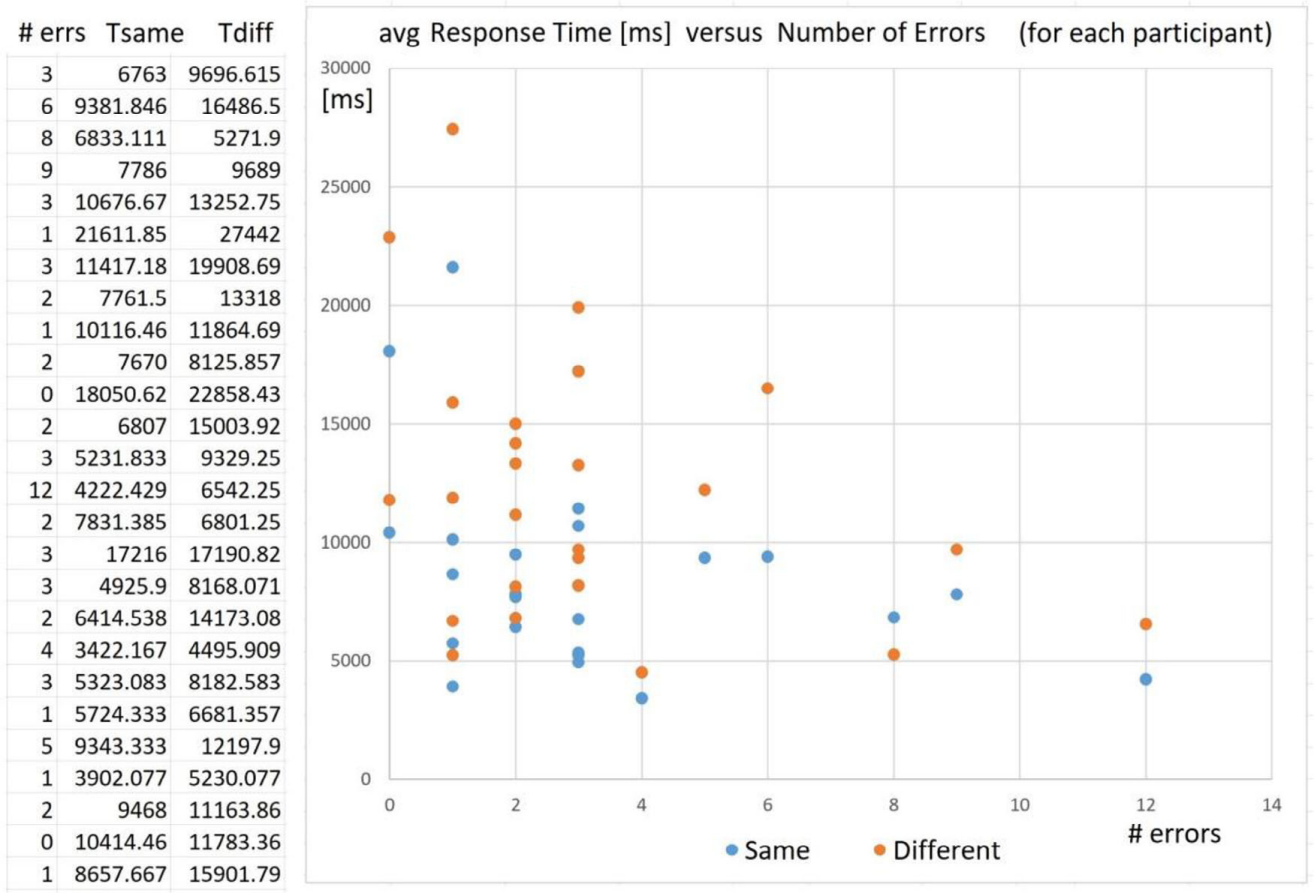
By comparing the raw response time with the number of error trials, we can demonstrate a classical speed‐accuracy tradeoff across the population of participants.

We begin with the analysis of the results of the test of baseline 3D spatial reasoning from 2D images. The table on the left of Figure 8 shows the number of errors made for each participant, along with the mean time to report ‘Same’ correctly and the mean time to report ‘Different’ correctly. Across all trials, some participants are very accurate, but tend to have longer task time, while some participants are faster (shorter task time), but tend to have more errors, as shown in Figure 9.

We can also make use of this classical paradigm to test a hypothesis about whether the task of performing a 3D task on the basis of 2D images is cognitive; involving top‐down spatial reasoning (an alternative hypothesis to what Shepard and Metzler originally proposed [11]). Many researchers propose that the brain has a mysterious functionality that allows 2D/3D ‘analogical’ representations to be rotated ‘in the head.’ The postulated theory says something to the effect of seeing two objects, somehow ‘rotating’ them so that they overlap, and then checking to see if they are the same or different. One problem with this theory is that no suggestion has been made as to *which direction* the 3D representation should be rotated in order to check (and you would first need to know in which way they were different in order to know which way to rotate them to check, “so, as they say… Catch‐22”). Aside from that, this analogical theory would predict that once the check has been made, the response at that time would either be the same or different, and so the amount of time needed to say ‘same’ or ‘different’ would be identical.

Conversely, for the top‐down spatial reasoning account, participants would examine the two side‐by‐side images that represent 3D structures. On this basis, they would deductively (top‐down) check parts of the structures to see if there was any reason that they were different. Notice that once there is any evidence that the shapes are different, then the participant can respond ‘different,’ whereas if the two objects are the same, then the participant must terminate the checking process on their own. In other words, at some point they would realize that with no evidence for a difference, that the objects must be ‘same.’ Accordingly, this alternate hypothesis would predict that the reaction time to say ‘same’ would be longer than ‘different.’

**FIGURE 10:**
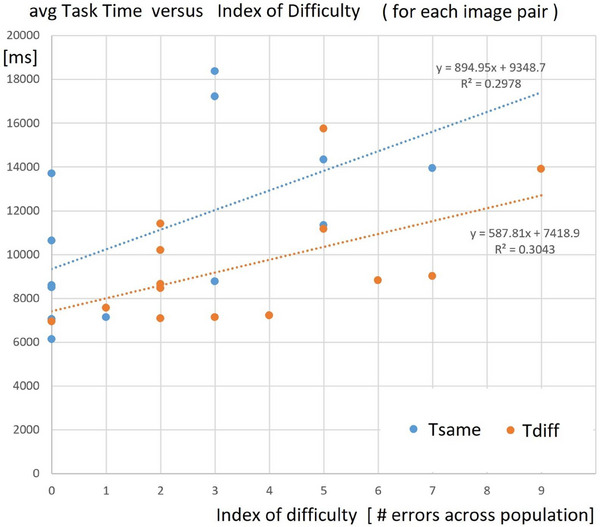
As a function of the index of difficulty of the stimulus pair, there is a significant difference between ‘same’ and ‘different’ trials, which is inconsistent with the classical ‘mental rotation’ interpretation. Instead, a top‐down cognitive spatial reasoning interpretation is implicated.

The results in Figure 10 show the reaction time, averaged across all participants, as a function of a raw measure of the index of difficulty for each trial pair. (The raw measure is simply the number of errors made across the population of participants—some image pairs were never mistaken by any of the participants, and some image pairs are so difficult that as many as nine participants judged them as same/different incorrectly). This figure shows that the reaction time is indeed a function of the index of difficulty, as is to be expected. But in addition, we see that the correlation is different for ‘same’ versus ‘different trials! Saying ‘Different’ is on average at least 2 s different from saying ‘Same.’ One observation afforded by this analysis is the response bias among participants: 53 times they say ‘same’ but the pairs are different, and 29 times they say ‘different’ when the pairs are the same. In other words, participants are biased to say “same,” even though the stimulus pairs are balanced evenly. Nevertheless, the difference shown here is problematic for an account based on a putative ‘mental rotation’ capacity, and it provides more converging evidence for a top–down cognitive account of spatial reasoning skills. This will have implications for the way that we recommend training surgical skills for PGY1 residents.

One motivation for studying user behaviour from a psychophysical perspective is that it can allow a focus on the modes of teaching and learning that are most effective. For example, from a cognitive science perspective, the behaviour of the user is modeled in a top‐down fashion. Accordingly, the teaching and learning mode should be one of didactic training, debriefing, and mentorship (rather than low‐level, bottom‐up repetitive training and perceptual‐motor adaptation).

### 4.4 A review of the construct tested with this simulator: ‘EVD trajectory planning skill’

There is no broader division in our literature, for approaches to clinical skills training and assessment than the divide between approaches that prescribe the use of subjective scores versus objective metrics. Furthermore, because earlier sections of this paper have raised a discussion point about the use of the terms ‘face validity,’ ‘content validity,’ and the *elusive* ‘construct validity,’ we offer a controversial point that might help to refocus efforts, at least for discussion amongst participants in this AECAI workshop. First, on the one hand, ‘objective metrics’ seem to be desirable because, if well‐formulated, they remove the ubiquitous cognitive biases that are typically manifest for both the experimenter and the subject of the experiment. On the other hand, in order to formulate an objective metric, many ideological assumptions inevitably must be asserted—so many, it would seem—that the desire to control the parameters of the simulated environment leads to an explosion of experimental conditions that make their presentation infeasible (this is the burden that psychophysical investigations bear in the classical literature of experimental psychology and cognitive science). In contrast, when trying to assess the performance of a skill in a reasonably realistic variety of possible scenarios, the assessment of performance is subject to the ill‐posed nature of such scenarios, in the sense that there may be several possible reasonable actions. Accordingly, the only recourse for evaluation is to appeal to the subjective scoring provided by a domain expert [12].

It does not seem to stretch the imagination of a reasonable researcher to propose that the distinction between ‘objective metrics’ and ‘subjective scores’ is exactly the distinction between the irreconcilable difference in the vValidity literature [13] between ‘internal validity’ and ‘external validity.’ As such, we must face an inevitable trade‐off, which has always been acknowledged in experimental psychology and cognitive science: The more internal validity ‘baked’ into your experimental paradigm, the more objective your measures can be. And, on the other hand, the more ‘external validity’ attributed to your experimental paradigm, the more inevitability will be the need to recourse to subjective scores. Accordingly, we have an inescapable trade‐off that needs to be acknowledged face‐on. You cannot have both! Your experimental paradigm cannot feasibly have both ‘high internal validity’ and ‘high external validity.’ Your AR‐VR‐based simulator cannot be ‘anatomically faithful’ and at the same time control for the index of difficulty induced by ‘anatomical variations’ across your training set [14, 15, 16].

In order to try to establish ‘construct validity,’’ there must be iterations between the two paradigmatic styles (not to mention the often missed step of first identifying what you mean by your ‘construct’ before you try to validate whether your measures are able to provide evidence of such). Iterations between these two styles of experimental paradigm are necessary if they are to establish converging evidence that they are valid measures of the elusive ‘construct.’ Only through effortful convergence and iterative design and evaluation cycles can the holy grail of ‘construct validity’ be established. (You certainly cannot establish ‘construct validity’ by showing that your scores correlate with the PGY rankings of your participant pool. That is merely ‘criterion validity,’ and should be acknowledged as such: an initial indicator that shows a rough, but necessary, sensitivity of your measures.)

## 5 CONCLUSIONS AND DISCUSSION

**FIGURE 11:**
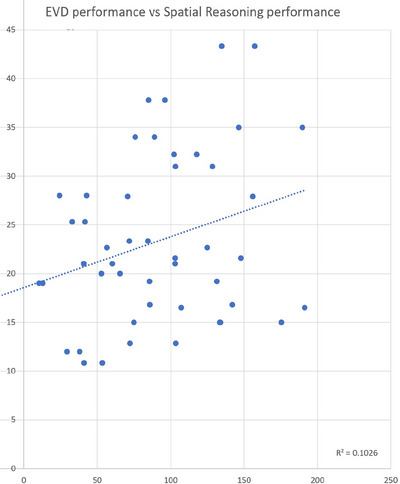
The scores on EVD performance are correlated with the participants’ objective scores on the spatial reasoning task.

Our main intent for this paper has been to foster a renewed discussion about the importance of the domain of the ‘evaluation of clinical simulators for procedure training.’ Our first comment on this topic, which will not be controversial, is that the evaluations should be as objective as possible—and therefore metrics of performance need to be formulated according to effortful and descriptive information‐processing models of the task or procedure that is being trained within a clinical curriculum (as demonstrated in Figure 11). While that may seem trite and obvious, many reports in the literature ignore this. Furthermore, by adopting this as a principle, one can still be led to some difficult questions: For example, if your trainees are performing a procedure in which the outcome does not depend on ‘path length,’ then why would you propose measuring ‘path length’ just because another study measured it as an explicit constraint on their task? Likewise, if the performance of a procedure does not depend on the force with which you grasp a tool, then why include that as a measure of performance on the task? If your performance on the task can be measured on the basis of the speed and accuracy performance of the task, then why measure the characteristics of the heartbeat or the conductance of the sweaty hands of the participant? Or the ‘hot spots’ of their eyemovements?

To be sure, each of these measures may (if you are lucky) correlate with the clinician's overall performance, but so might their performance correlate with the proportion of grey hairs on their head, or the price of their car in the parking garage. Each of these measures will probably correlate with performance. But at the end of the day, the only real measure of performance will be to decompose the procedure into phases, and the phases into tasks, and the tasks into sub‐tasks, until the leaf nodes are either ‘targeting’ tasks or ‘decision‐theoretic choices.’ At that point, each phase can either be evaluated objectively in terms of speed and accuracy, or (inversely) the task time and error rate. Yes, now those are controversial stances—so let us discuss.

## AUTHOR CONTRIBUTIONS

**Roy Eagleson**: Conceptualization; data curation; formal analysis; funding acquisition; investigation; methodology; project administration; resources; software; supervision; validation; visualization; writing—original draft; writing—review and editing. **Denis Kikinov**: Investigation; resources; software. **Liam Bilbie**: Investigation; resources; software. **Sandrine de Ribaupierre**: Conceptualization; data curation; funding acquisition; investigation; methodology; project administration; resources; supervision; validation; visualization; writing—original draft; writing—review and editing.

## CONFLICT OF INTEREST STATEMENT

The authors declare no conflicts of interest.

## Data Availability

The data that support the findings of this study are available from the corresponding author upon reasonable request.

## References

[1] Eagleson, R. , de Ribaupierre, S. , King, S. , Stroulia, E. : Medical education through virtual worlds: The HLTHSIM project. Stud. Health Technol. Inf. 163, 180–184 (2011) 21335785

[2] De Ribaupierre, S. , Kapralos, B. , Haji, F. , Stroulia, E. , Dubrowski, A. , Eagleson, R. : Healthcare training enhancement through virtual reality and serious games. In: Virtual, Augmented Reality and Serious Games for Healthcare, pp. 9–27. Springer, Berlin, Heidelberg (2014)

[3] Kersten‐Oertel, M. , Gerard, I.J. , Drouin, S. , Mok, K. , Sirhan, D. , Sinclair, D.S. , Collins, D.L. : Augmented reality for specific neurovascular surgical tasks. In: Proceedinngs of Augmented Environments for Computer‐Assisted Interventions: 10th International Workshop, AE‐CAI 2015, Held in Conjunction with MICCAI 2015, pp. 92‐103. Springer International Publishing, Berlin (2015)

[4] Haji, F.A. , Clarke, D.B. , O'Kelly, C. : How to build a national boot camp: Experience from the Canadian Neurosurgical Rookie Camp. Paper presented at the 2013 International Conference on Residency Education, ICRE, Royal College of Physicians and Surgeons, Calgary, Canada 26–28 September 2013

[5] Haji, F.A. , Clarke, D.B. , Matte, M.C. , Brandman, D.M. , Brien, S. , de Ribaupierre, S. , O'Kelly, C. , Christie, S. , McDonald, P.J. , Kulkarni, A.V. , Walling, S. , MacLeod, A. : Teaching for the Transition: the Canadian Neurosurgery Rookie Camp. Can. J. Neurol. Sci. 42, 25–33 (2015) 25573052 10.1017/cjn.2014.124

[6] Dahlstrom, N. , Dekker, S. , van Winsen, R. , Nyce, N. : Fidelity and validity of simulator training. Theor. Issues Ergon. Sci. 10(4), 305–314 (2008)

[7] Nguyen, N. , Eagleson, R. , Boulton, M. , de Ribaupierre, S. : Realism, criterion validity, and training capability of simulated diagnostic cerebral angiography. Stud. Health. Technol. Inf. 196, 297–303 (2014) 24732526

[8] Roy, E. , Leo, J. : Verification, evaluation, and validation: Which, how, and why, in augmented reality system design. J. Imaging 9(2), 20 (2023) 36826939 10.3390/jimaging9020020 PMC9965271

[9] Yaniv, Z. , Linte, C.A. : Applications of augmented reality in the operating room. Fundamentals of Wearable Computers and Augmented Reality, pp. 485–518. CRC Press Taylor and Francis Group, Boca Raton FL (2016)

[10] Armstrong, R. , Noltie, D. , Eagleson, R. , de Ribaupierre, S. : An examination of metrics for a simulated ventriculostomy part‐task. Stud. Health Technol. Inf. 220, 29–32 (2016) 27046549

[11] Shepard, R. , Metzler, J. : Mental rotation of three‐dimensional objects. Science 171(3972), 701–703 (1971) 5540314 10.1126/science.171.3972.701

[12] Neumuth, T. , Loebe, F. , Jannin, P. : Similarity metrics for surgical Process models. Artif. Intell. Med. 54(1), 15–27 (2012) 22056273 10.1016/j.artmed.2011.10.001

[13] Cronbach, L.J. , Meehl, P.E. : Construct validity in psychological tests. Psychol. Bull. 52, 281–302 (1955) 13245896 10.1037/h0040957

[14] Abhari, K. , Baxter, J.S.H. , Chen, E.S. , Khan, A.R. , Wedlake, C. , Peters, T. , Eagleson, R. , de Ribaupierre, S. : The role of augmented reality in training the planning of brain tumor resection. In: Augmented Reality Environments for Medical Imaging and Computer‐Assisted Interventions: 6th International Workshop, MIAR 2013 and 8th International Workshop, AE‐CAI 2013. Held in Conjunction with MICCAI, pp. 241‐248. Springer, Berlin (2013)

[15] de Ribaupierre, S. , Armstrong, R. , Noltie, D. , Kramers, M. , Eagleson, R. : VR and AR simulator for neurosurgical training. In: Proceedings of IEEE Virtual Reality (VR), pp. 147–148. IEEE, Piscataway, NJ (2015)

[16] de Ribaupierre, S. , Eagleson, R. : Challenges for the usability of AR and VR for clinical neurosurgical procedures. Healthcare Technol. Lett. 4(5), 151–151 (2017) 10.1049/htl.2017.0077 PMC5683198 29184655

